# Supplementary material for: Multitask learning over shared subspaces
Source: PLoS Comput Biol. 2021 Jul 6;17(7):e1009092. doi: 10.1371/journal.pcbi.1009092 (PMC8284664; doi:10.1371/journal.pcbi.1009092)
Supplement: S1 Text — (PDF) [file pcbi.1009092.s001.pdf]

Supporting Information

1 Neural Network Model

1.1 Likelihood Gradient

This section describes the backpropagation algorithm for computing the gradient of the likelihood function with respect to the network parameters. It is therefore very similar to most textbook expositions [1] but is specific to our architecture (three layers, partitioned structure in the output networks). For each of $n = 1..N$ output networks we have

$$\begin{aligned} \tilde{\delta}^n_{tk} &\equiv \frac{dL_t}{d\tilde{a}^n_t} \\ \tilde{\delta}^n_{t1} &= r_t - v^n_{t1} \\ \tilde{\delta}^n_{t2} &= -(r_t - v^n_{t2}) \\ \frac{dL_t}{d\tilde{w}^n_j} &= \tilde{\delta}^n_{tk}\tilde{x}^n_{tj} \\ \frac{dL_t}{d\tilde{b}^n_k} &= \tilde{\delta}^n_{tk} \end{aligned} \tag{1}$$

The signs in the above equation denote, for example, that output weights are increased for rewards under action $k = 1$, but decreased under $k = 2$. The likelihood gradients or "errors", $\tilde{\delta}$, are "backpropagated" to the previous layer and concatenated as

$$\begin{aligned} \tilde{g}^n_{tj} &= f'_2(\tilde{x}^n_{tj})\tilde{w}^n_j\tilde{\delta}^n_{tk} \\ \delta^2_t &= [\tilde{g}^1_t; \tilde{g}^2_t; ...; \tilde{g}^N_t] \end{aligned} \tag{2}$$

where the last equation runs over all j (layer 2) units in each output network, and $f'_2()$ is the derivative of the second-layer activation function. For $k = 1..H_2$ nodes in the second hidden layer we have

$$\begin{aligned} \delta^2_{tk} &\equiv \frac{dL_t}{da^2_{tk}} \\ \frac{dL_t}{dW^2_{kj}} &= \delta^2_{tk}x^1_{tj} \\ \frac{dL_t}{db^2_k} &= \delta^2_{tk} \end{aligned} \tag{3}$$

where the errors, δ, are back-propagated to the previous layer. For $k = 1..H_1$ nodes in the first hidden layer we have

$$\begin{aligned} \delta^1_{tk} &\equiv \frac{dL_t}{da^1_{tk}} \\ &= f'_1(x^1_{tk})\sum_{j=1}^{H_2} W^2_{kj}\delta^2_{tj} \end{aligned} \tag{4}$$

Gradients of first layer weights are then

$$\begin{aligned} \frac{dL_t}{dW^1_{kj}} &= \delta^1_{tk} u_{tj} \\ \frac{dL_t}{db^1_k} &= \delta^1_{tk} \end{aligned} \tag{5}$$

The derivatives of the first and second layer activation functions, $f'_1(x)$ and $f'_2(x)$ are $x(1-x)$ for sigmoids, $[x > 0]$ for RELUs, $\Phi(x) + x\phi(x)$ for GELUs (where ϕ is the probability density function of the Gaussian distribution), $\sigma(x)$ for SoftPlus and 1 for linear units. We can write a gradient vector using the Pack function defined in the main text. That is,

$$\begin{aligned} g_t &\equiv \frac{dL_t}{d\theta} \\ &= \mathsf{Pack}\left[\frac{dL_t}{dW}, \frac{dL_t}{db}\right] \end{aligned} \tag{6}$$

To compute the Hessian matrix (curvature) we need the output sensitivity

$$\eta_t \equiv \frac{d\tilde{a}^n_t}{d\theta} \tag{7}$$

This follows from the chain rule as

$$\begin{aligned} \frac{dL_t}{d\theta_i} &= \frac{dL_t}{d\tilde{a}^n_t}\frac{d\tilde{a}^n_t}{d\theta_i} \\ g_t(i) &= \tilde{\delta}^n_{tk}\eta_t(i) \end{aligned} \tag{8}$$

Hence, $\eta_t = g_t/\tilde{\delta}^n_{tk}$.

2 Further Analyses of Behavioural Data

Learning Accuracy Across Mappings

Participants performed better in the linearly separable tasks (addition and subtraction in Task 2) compared to the nonlinearly separable tasks. A one tail, dependent sample t-test revealed the effect to be significant (mean linear = 0.735, mean nonlinear=0.589, $t(158) = -9.234$, $p < 0.001$). However, this result is biased by both order and subspace effects. Collapsing across Tasks 1 and 2, we found no significant effect of subspace (addition versus subtraction) with mean addition = 0.667, mean subtraction = 0.657 ($t(158) = 0.491$,$p = 0.624$).

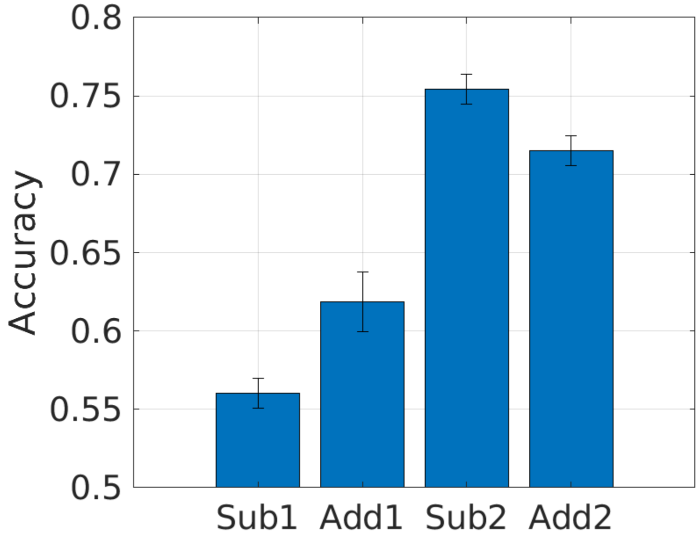

Fig A. Effect of Mapping on Accuracy. The plot shows the mean accuracy for each mapping with error bars indicating the standard error of the mean.

2.1 Within- versus Between-Block Learning

To identify at which time scale it would be most appropriate to run Sequential Bayesian learning within a learning task, we tested to see how much learning takes place within versus between blocks as subjects learn the first task.

We first look at learning within blocks by comparing the average accuracy increase (averaged over all 80 subjects) from the first 25 to the last 25 trials. This increase was significant (one-sided) for all five learning blocks (paired t-tests, average increase and two-sided p-values for Block 1: Mean=0.038, p=0.029; Block 2: Mean=-0.036, p=0.021; Block 3: Mean=0.051, p=0.00004; Block 4: Mean=0.023, p=0.100; Block 5: Mean=0.033, p=0.029).

We now look at learning between blocks by comparing the average accuracy increase (proportion correctly classified) from one block of trials to the next. This increase was significant for the first 3 transitions between blocks (paired t-tests, average increase and two-sided p-values for Block 1 to Block 2: Mean=0.033, p=0.021; Block 2 to Block 3: Mean=0.031, p=0.028; Block 3 to Block 4: Mean=0.038, p=0.001; Block 4 to Block 5: Mean=-0.003, p=0.824). Note that no significant learning takes place in the last transition.

We then looked to see if the increase between blocks was significantly greater than within - it was not (paired t-test, Mean Between = 0.025, Mean Within = 0.0218, p=0.17). Finally, we compared the first 25 trials of one block to the last 25 trials of the previous block to find out if there was an increase during the break between blocks. This difference, Mean Between = 0.0065, was less than Mean Within = 0.0218 (paired t-test, p=0.17) so there is no benefit of the break (if anything there's a startup cost).

Overall, this analysis shows that learning takes place within blocks and there is no beneficial effect of breaks. This motivated us to choose a block size of 25 trials for the simulations of within task learning (see subsection on Sequential Bayesian Learning over Blocks and Tasks in the Results section). Each point in Fig 6. (right panel) shows mean accuracy averaged over two subsequent blocks (i.e. 50 trials).

2.2 Self-Reports on Task 1

We first define a declarative learner as a subject who correctly declared their strategy for the first task. This was based on the participant's response to the open-ended question posed after each task: "How did you approach the task?". A correct declaration of strategy was inferred if their verbal report contained the following phrase (i) for Sub1 "if the number of pie slices is the same/similar in both pies, then choose Sun" or , (ii) Add1 "if the total number of slices on the left and right makes a complete pie, then choose Sun". Phrases that were deemed logically or semantically equivalent were also assessed positively. All assessments were made by author NM.

A two-way between-subjects ANOVA with dependent variable accuracy and independent factors of declaration (declared/not declared) and subspace (same/different) showed a main effect of subspace (F(1,76)=7.66,p=0.007), no main effect of declaration (F(1,76)=2.7, p=0.104) and a significant declaration by subspace interaction (F(1,76)=7.51, p=0.008). Note that the size of the subspace effect in the declarative group is 12 per cent, much larger than the subspace effect computed over all subjects (4.3 per cent - see main text). For the declarative learners in the same subspace group (N=14) the mean accuracy in task 2 is 82 per cent, whereas for declarative learners in the different subspace group (N=15) the mean accuracy in task 2 is only 70 per cent. For the non-declarative learners the corresponding figures are both 72 per cent.

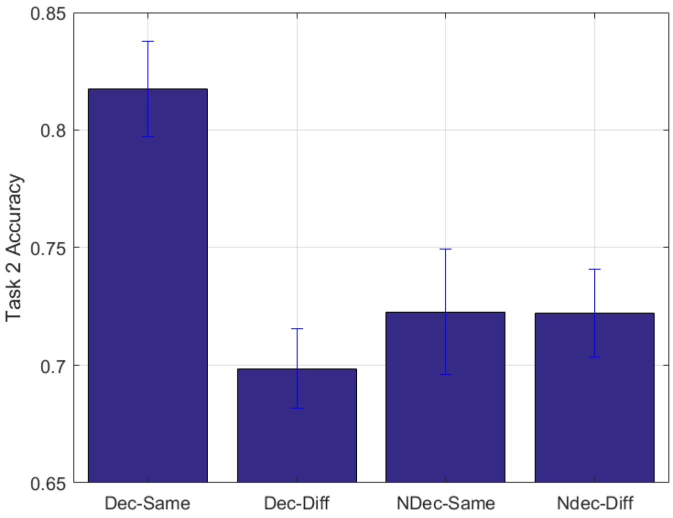

Fig B. Declaration by Subspace Interaction. The left two bars are for declarative learners and the right two for non-declarative.

However, we cannot conclude from this analysis that the subspace effect is driven by declarative learners. This is because declarative learners also performed well on the first task. On the first task overall, average correct rates were 66 and 55 per cent for participants who declared or could not declare their strategy ($t(78) = 5.45, p < 10^{-6}$). On the last block of the first task, these correct rates were 76 and 56 per cent ($t(78) = 6.22, p < 10^{-7}$). We then defined a "good learner" as being in the group of those 29 subjects with highest performance in the last block of the first task (we chose 29 subjects to match the number of declarative learners). By this definition 19 of the 29 good learners also declared. A two-way between-subjects ANOVA with dependent variable accuracy and independent factors of learner (good/bad) and subspace (same

different) showed a main effect of subspace (F(1,76)=5.46,p=0.022), a main effect of learner (F(1,76)=6.32, p=0.014) and a significant learner by subspace interaction (F(1,76)=4.01, p=0.049). For good learners in the same subspace group (N=16) the mean accuracy in task 2 is 81 per cent, whereas for good learners in the different subspace group (N=13) the mean acccuracy in task 2 is only 72 per cent. For bad learners the corresponding figures are 72 and 71 per cent.

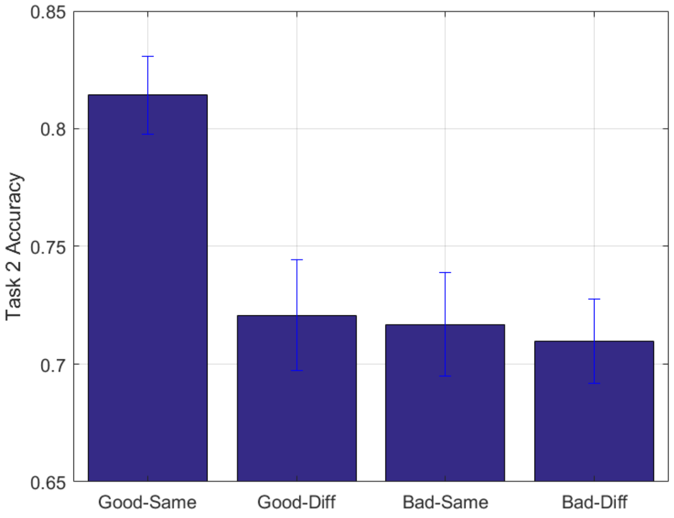

Fig C. Learner by Subspace Interaction. The left two bars are for good learners and the right two for bad learners.

But, again, we can't conclude that being a "good learner" drives the subspace effect as many of these subjects (19/29) also declared. To shed further light on this matter we ran a 3-way between-subjects ANOVA with factors of subspace, declaration and learner, and neither of the above two-way interactions (declaration by subspace and learner by subspace) were significant. Furthermore, we calculated the Variance Inflation Factor between these predictors, finding a moderate collinearity of VIF=1.715. We therefore cannot tell which of these factors is driving the subspace effect.

2.2.1 Self Reports on Task 1 and 2

More participants declared the correct strategy in the second task compared to the first one ($x^2(1) = 4.949$, $p = 0.026$). No difference was found between subtraction and addition conditions in task 1 ($x^2(1) = 2.650$, $p = 0.103$). Significantly more participants declared the subtraction strategy in task 2 compared to the addition one ($x^2(1) = 4.073$, $p = 0.043$).

	Subtraction	Addition
**Task 1**	11/40	18/40
**Task 2**	26/40	17/40

Table A. ***Declarations in task 1 and 2*** *The table shows a breakdown of the number of participants who declared the correct strategy in task 1 and task 2 as a function of Add/Sub subspace.*

References

1. Nabney I. NETLAB: Algorithms for Pattern Recognition. Springer; 2003.
