# Supplementary material for: Multitask learning over shared subspaces
Source: PLoS Comput Biol. 2021 Jul 6;17(7):e1009092. doi: 10.1371/journal.pcbi.1009092 (PMC8284664; doi:10.1371/journal.pcbi.1009092)
Supplement: Attachment — Submitted filename: Reviews.docx [file pcbi.1009092.s002.docx]

# Editor

Editor's comments:

The reviewers cover most of the key issues I noticed in the paper.

Q0.1. The one additional point I want to raise is that some of the statistical results are pretty weak (even with the liberal use of one-sided tests, some of the results are not very strong). Weak results by themselves are not problematic but in this case there is the risk of false positives and p-hacking, where a two-sided test is sometimes done first (revealing no significant effect), followed by a one-sided test (revealing a significant effect).

All p-values are now two-sided.

We have made some major changes to the paper. In response to reviewer 3 (Q 3.6) we have greatly reduced the number of behavioural data analyses we present. We have also completely revised the modelling work (see responses to Q3.8), and made many major additions to the text throughout the paper.

We’d like to thank the Editor and Reviewers for giving their time to review this paper.

Reviewer's Responses to Questions

**Comments to the Authors:**

# Reviewer 1

**Q1.0** Reviewer #1: In this paper, the authors show that humans can perform a new task better, at least initially, if the relevant features are the same as those from a previous task. Using computational modeling, they show that this effect be accounted for by a form of “soft parameter sharing“ (SPS): a technique employed in multitask learning, whereby the parameters for the new task are initialized with parameters from a previous task.

In particular, on each trial, the inputs u (u1 = # slices on pie 1, u2 = # slices on pie 2) are mapped onto latent features x by a linear transformation, x = Au. The features are then mapped onto outputs in potentially nonlinear ways. Two tasks are said to share the same subspace if the optimal A is the same (even if the mapping from features to outputs is different). The authors explore three computational alternatives for the way in which humans learn to perform the tasks:
- soft parameter sharing (SPS): A for task 2 is initialized with A from task 1, but is potentially overridden if performance is not good
- Hard parameter sharing (HPS): A for task 2 = A for task 1
- RBF: A = I for both tasks, i.e. x = u
They show that the behavior of SPS is most consistent with the human data.

Overall, I found the paper to be relatively well-written and clear. It is covering a very timely question: the ability of humans to learn new tasks so quickly is thought to rely on inductive biases from past experience, but the details of how this process unfolds and how it might be implemented in the brain are far from clear. Multitask learning provides a formal framework for acquiring such biases and modifying them with experience, and thus can naturally serve as a source of hypotheses of how the brain might be doing it. While on the surface, the result might not seem so surprising — if you give people a similar task, of course they do better — formalizing what “similar” means (same latent features, as in this paper, as opposed to e.g. same output mapping) and studying the nuances of how exactly they “do better” (e.g. better at first vs. throughout) are essential steps in testing hypotheses about the underlying computational process. Thus I think this paper is an important step towards elucidating the details of multitask learning in humans.

Thanks for the insightful summary and encouraging remarks.

I do have a few suggestions on what the author can do to improve it.

Q1.1 First, I think the authors should include a No Parameter Sharing (NPS) model, which is the same as SPS, except the A’s are not constrained to be similar across task. This seems like a plausible alternative, which will do better initially in the different subspace condition compared to SPS. Naturally, it will not have an advantage in the same-subspace condition, so ultimately it is a bit of a strawman model, however I think it is more plausible than the RBF model and I suspect some subjects might be relying on something like that (i.e., not transferring any knowledge and learning the new task from scratch).

The essence of the No Parameter Sharing model is now captured in the “Increased Capacity Model”, where a second hidden unit in the first layer is essentially free to take on new representations.

Q1.2 Second, I would consider adding a version like SPS but without adjusting the precision as in Eq 15. That is, just continual learning, as if you’re still getting trained on the same task even though you’re on task 2. Note that this is not the same as HPS, since A can still keep changing for task 2 (whereas for HPS it is fixed), it’s just that the rate at which it is changing is fixed. I think this simpler form of learning (which in fact might not even be considered multitask learning, as from the point of view of the agent, there’s just a single task) might account for the data as well as SPS.

SPS has now been removed from the paper. The procedure you propose is now implemented in both the Minimal and Increased Capacity Models.

Q1.3Third, I think the authors could do a better job of visualizing the links between human and model behavior. I recommend reorganizing the figures (and perhaps the exposition), such that each figure shows the same effect for humans, SPS, HPS, and NPS side-by-side, in the same way and with the same analysis. This would also consolidate the figures a little bit.

We now plot both the human and model-based learning trajectories in consecutive figures for direct comparison.

Minor:

**Q1.4** The notation is not very clear, and I recommend the authors explicitly define all the variables after each equation unless they have already been defined. E.g., equation 2: y, u, mu, etc are not defined. Or equation 3: what is phi? What is n? Some of these are defined only later or not at all. Also, the subscript notation is ambiguous: at first, u_t denotes trial, but then u_1 denotes the first component of u (line 113).

We have now defined each variable the first time it is introduced and have checked for inconsistencies in the use of subscripts and brackets.

**Q1.5** Line 121: did they learn a different task on the different day?

We have now added the text: “These two tasks were carried out on the same day in a 1 hour long experiment.”

**Q1.6** I would add subsections to the Value Network section explaining precisely how SPS, HPS, NPS, and RBF differ from the generic Eq 3, with separate equations. Right now, this information is interspersed throughout.

We have now replaced all of these models with new ones.

**Q1.7** The section on off-policy learning is confusing: isn’t the same agent learning and also performing the task?

You’re right – this was an incorrect use of terminology. We have now replaced this text (in the section on Model Likelihood) with

“This paper employs an offline learning approach (similar in concept to offline Reinforcement Learning (Levine and Fu, 2020)), in which data is stored in a memory buffer.”

**Q1.8** The likelihood function P(R|A) in Eq 6 is not defined anywhere. Also, the notation is a bit ambiguous, as m is already used to denote the RBF centers.

We have now removed “m” as we no longer have RBF centres (or consider rows of the subspace matrix). A new subsection on Model Likelihood now defines p(R|A).

**Q1.9** What are the error bars in figure 5 and 7?

The error bars are the standard error of the mean which we now indicate in the caption of figure 5 (figure 7 has been removed).

**Q1.10** What is adjusted data in figure 6?

This figure has been updated (now presented as figure 4) without this terminology. There had in fact been no “adjustment” – this was just standard text produced by a particular Matlab function.

**Q1.11** Line 334 and 337: F statistic and df’s

These have been removed now.

**Q1.12** Figure 8 and the corresponding text seem redundant with figure 6; just a different way of visualizing the same result

This figure has been removed.

**Q1.13** Figure 9 seems to be contradicting figure 7 – this itself is an interesting result which should be further looked into and perhaps investigated using computational modeling.

We don’t think there was a contradiction here. The top curve in figure 7 was for all learners in the same subspace group. In figure 9 this “top curve data” was then broken down into “good” versus “bad” learners. We have, however, for other reasons (see our responses to Q3.6) removed both of these figures.

**Q1.14** Line 408: what drives the difference between good and bad learners? Is it just the stochasticity in the parameter optimization process? Or the initialization? Or the training?

In the neural net simulations this was driven by stochastic differences in the initialisation of parameters. The training was deterministic. Though we no longer make a distinction between good and bad learners we now write (in the subsection on “MAP Estimation”):

“ All \theta values are initialised by sampling from the prior. This is a stochastic process which leads to different results on each simulation run. Other than this sampling process, the optimisation is deterministic.”

**Q1.15** Relatedly, on line 413, what drives this effect? Is it that the better learners learned the correct feature mapping, or are they just better at learning overall? The benefit of having a computational model is that you can easily answer those questions, whereas it is difficult to answer them for the participants directly.

Due to the stochasticity in the initialisations used in the multistart optimisation (see Q1.14), task 1 was learnt better for some simulation runs than others. Using a median split over accuracy in the last block of the first task, this allowed some runs to be declared as from “good learners”, others from “bad”. So good learners are better at learning the appropriate feature mapping in task 1.

However, in the new version of the paper we make no mention of good versus bad learners (see our responses to Q3.6).

**Q1.16** Relatedly, line 421 – This differs from the human data and bears further discussion, also investigating in more detail what in the computational model drives this difference.

The new version of the paper does not contain the SPS model so we make no direct comment on this in the new version. However, the new discussion section on “Discrepancies between model and behavioural data” does make reference to this under point 3. Essentially, this effect is apparent in the subjects who initially did Sub1 as the first task, but not those who did Add1. Due to this inconsistency ,our opinion is this is not robust enough to warrant modelling.

**Q1.17** Relatedly, line 436 – what is driving this difference?

Because the HPS model uses the wrong prior for task 2, and cannot change it, it has no mechanism for recovery. Whereas the SPS model can dilute the wrong prior. In the new version of the paper, however, we do not discuss the SPS and HPS models.

**Q1.18** Line 469 and the following paragraph – Not sure I get distinction between features and subspaces, as defined here. Any subspace can be defined by a basis set of features, so the two seem isomorphic. I suppose what the authors are getting at is that when the dimension of the subspace is 1 (and hence it can be described by a single feature), there might be simpler strategies that subjects could be relying on?

Yes, this all seems a bit redundant. We have now removed this paragraph.

# Reviewer 2

**Q2.0** Menghi et al. present results from a behavioral task that assesses humans’ ability to exploit shared structure between different reward environments (“tasks”). They also develop a computational model of how an agent may exploit this shared structure based on the ML framework of Multitask Learning.
The paper claims that:
* The task is a novel way to study transfer learning
* The model somewhat matches how humans learn
* Shared subspaces are a useful framework for thinking about multitask learning
Overall I am enthusiastic about the approach, but found that the paper could benefit from (1) More focused claims, and relating the findings to several strands in the literature that have already examined transfer learning (albeit under slightly different names); (2) Deeper analysis of ways in which the model succeeds or fails in capturing various aspects of how humans behave in this task.

This is a fair and accurate summary and we hope that our revisions address the deficiencies.

Major comments:

**Q2.1** It is stated only later, but the authors hypothesized that learning is easier for tasks that share a common subspace. This finding on its own is not particularly surprising. As the authors point out in the intro, we know from previous studies of structure learning that people take advantage of shared structure across tasks (e.g. https://www.jneurosci.org/content/35/6/2407, https://www.biorxiv.org/content/10.1101/815332v1 and <https://journals.plos.org/ploscompbiol/article?id=10.1371/journal.pcbi.1006116>).

We now discuss these papers in a new “Structure Learning” subsection in the Introduction.

**Q2.2** That said, the authors have an interesting design which could be leveraged to ask specific questions about how humans actually manage to do this. For example, what is going on with the obvious difference in accuracy between Task 1 and Task 2? Is the main difference that Task 1 requires learning a non-linearity? If so, that is interesting! There is a long-standing debate in the category learning literature about whether and how humans can learn non-separable (NS) categories (see https://psycnet.apa.org/record/2011-17802-001 and https://link.springer.com/article/10.3758/s13421-019-00972-y). One idea is that humans shift from elemental (feature-based) to configural (object-based) representations as they learn (see https://www.cell.com/trends/cognitive-sciences/fulltext/S1364-6613(19)30036-1 and https://www.nature.com/articles/s41467-017-01874-w). Do the authors see evidence of this in their data? Does the model they present display such a shift in representation across learning blocks? How does linear separability interact with humans’ ability to exploit shared structure? And to what extent does learning the NS task first affect parameter sharing?

To be clear, I am not necessarily suggesting that authors answer all these questions in the paper, just pointing out a direction they could take to see how their model can speak to some of the open questions in the literature.

We have now included a new section in the Introduction on “Elemental and Configural Learning”, and when introducing the mappings used in the paper discuss how they fit in with this. We also include an analysis of accuracy of learning linearly separable versus non-linearly separable mappings (see Appendix C), with its own subsection in the discussion (“Learning accuracy across mappings”).

**Q2.3** Second, it really wasn’t clear to me just how well the model captures key aspects of human data.

In Figure 7, we see that subjects exploit structure, which manifests as a performance gain early during Task 2 and gradually increases over time. This pattern suggests that people learned some kind of higher order rule, such as the one authors suggested at the bottom of page 4. But in Figures 10 and 11, which show us what the model is doing, it looks like in both conditions the model starts Task 2 much closer to chance and the improvement is then sudden and stays constant. Both of these patterns would be picked up by an ANOVA, but they suggest qualitatively different learning dynamics. So what is going on here? In terms of presentation, it would really help to add the human plot next to Figures 10 and 11 to make the comparison clear.

We now show the learning trajectories from both the behavioural data and the model in adjacent figures. There are clear differences and these are briefly referred to in the results section and examined at greater length in the Discussion (see “Discrepancies between model and behavioural data”).

**Q2.4** Similarly, even in the different subspaces condition, it looks like the model starts much lower than humans do (45% performance in Fig. 11 vs. 65% performance in Fig. 7).

Yes, this is quite correct. Even in the new model (see responses to Q3.8) this is indeed the case. In the discussion we now attribute this to learning from blocks of trials rather than single trials – for the first block of data from Task 2, model performance will therefore be close to chance (as the model has not yet had an opportunity to tune model parameters).

**Q2.5** It’s striking that Hard Parameter Sharing fails so spectacularly when tasks do not share a subspace. SPS seems critical but maybe even too good compared to humans? Is it possible to vary the degree of “sharedness”, and if so, what would the optimal level be for this task?

We now try to broadly match the model accuracy on Task 1 with accuracies from behavioural data. We have done this by tuning the maximum number of starts in the multistart optimisation process that runs over each block.

**Q2.6** It could be that some of these differences stem from reporting Accuracy for humans and Average Rewards for the model, but it’s not possible to know without a side-by-side comparison and directly simulating actions by the trained model.

We do simulate actions from the trained model. This is now explained more clearly in the first paragraph of the results sections on “Sequential Bayesian Learning over Tasks” and “Sequential Bayesian Learning over Blocks and Tasks”.

**Q2.7** Were subjects explicitly instructed that the second task was different? Or was the difference only in the framing (sun/rain vs. heads/tails)? This seems important, as the network is strongly encouraged to separate by allowing it knowledge of the task label.

Yes, they were. We have now added the following sentence to the Experimental Design section: “Subjects were explicitly instructed that the mapping in the second task was different”.

**Q2.8** Finally, the authors might want to check out and connect to the work of several authors which is directly relevant for what they are trying to do here:
Wang et al.: https://www.nature.com/articles/s41593-018-0147-8
Musslick and Cohen: https://cogsci.mindmodeling.org/2019/papers/0161/index.html

Thanks for these references. We have added a subsection to the Introduction to briefly discuss these papers (“Neural Network Models”).

Minor comments:

**Q2.9** I found the training procedure section unclear: how does the NN training interact with sequential Bayes? Do you solve for the MAP and then use that as a training objective? And how does this interact with the log likelihood of *reward*?

Apologies for not presenting this more clearly – we have now included more technical sections in the main body of the paper with clear labels such as “Model Likelihood”, “MAP Estimation” and more text in the “Sequential Bayesian Inference” section describing how it all fits together. Essentially, (i) find MAP estimates on the new data block, (ii) find the posterior precisions (from Laplace approx.), (iii) these posteriors become the priors for the next iteration, (iv) go to (i). The whole thing together is Sequential Bayesian Inference.

**Q2.10** Can participants verbally articulate learning some of the rules? That datapoint might be another useful one in determining what is actually being learned

Participants were probed about their knowledge at the end of each task. We asked them to describe the way they approached the task. This is explained at the end of the “Procedure” section. More participants were able to declare the correct strategy in the linearly separable task compared to the nonlinearly separable one. Among the linearly separable tasks, more participants declared the subtraction than addition task (i.e. Sub2). Data points are reported and analyzed in the final paragraph of Appendix C.2.

**Q2.11** The Fig. 2 caption brought a lot of clarity regarding how subspaces were operationalized. Consider adding some version of this text to the “S-R mapping section” as a preamble. E.g. “Subspaces were operationalized by defining a common feature that, when represented, reduced the task to a rule. In one case this was the sum, in the other the subtraction… “ — and only then present the equation

We have now added such a sentence to the Stimulus Reward Maps section.

**Q2.12** Please take a pass through the paper and clarify notation as much as possible. Some examples:
* In equation 2, what are u, mu and W? These are only introduced later...
* In equation 3, v and h are not displayed in Fig. 3

Apologies for the carelessness here – we have now been through the paper again and defined variables when they first appear.

**Q2.13** Table 1 is helpful in clarifying the design; consider adding columns specifying: the number of subjects, the type of task (weather or coin) and the break

We have now added a number of subjects column, and explain in the table caption that “for all subjects, Task 1 was presented as a weather prediction task and Task 2 as a coin prediction task”.

**Q2.14** Stimulus-Reward Maps or “Value Functions”?
Correct terminology would be “Reward Function”, as it defines the reward the participant would get in a particular state contingent on action

We now use the term Reward Function rather than Value Function.

**Q2.15** Figures 10 and 11 have the wrong x-ticks (should be 1:10)

The corresponding figures in the revised version (Figs 6 and 7) have x-ticks from 1 to 10.

# Reviewer 3

Reviewer #3: REVIEW OF “MULTITASK LEARNING OVER SHARED SUBSPACES”


Summary

**Q3.0** This study presents an experiment on transfer between tasks with a
shared or non-shared feature subspace, as well as a model with “soft
parameter sharing” that can flexibly transfer a previously learnt
representation or not. The model is compared with a version which forces
“hard parameter sharing” (always transferring a representation) and
another model (RBF) which does not transfer. The experiment shows a
benefit in performance when tasks share a subspace, and the modeling
results show that only the soft parameter-sharing model shows this
benefit.

This is a fair summary.

Evaluation

**Q3.1** The manuscript is generally well-written and clear and the authors
clearly understand the models they developed (and how to make them
work). I find the results of the experiment interesting. Looking at
Figure 2, it doesn’t seem obvious why sharing an additive or subtractive
way to combine the cues would provide a benefit, given the different
ways in which this combined feature is mapped to value. Aspects of the
model are interesting too (e.g., combining Bayesian inference with
neural network models). I didn’t find the comparison between the models
that interesting though. Simulating models that either force complete
transfer or no transfer at all will obviously give the result of
(failed) transfer vs no transfer. As the models are not fit to
participants’ behaviour, the potentially interesting question whether
soft parameter sharing also best describes people in the same subspace
conditions remains unanswered. The RBF model with no transfer further
differs in many respect to the other two models and hence a clear
comparison is difficult. It seems easy enough to implement a model of
the same form as the other two with no transfer (i.e., simply resetting
the prior at task 2 to the same one at task 1), and I wonder why the
authors didn’t include this version.

We have now removed the RBF model from the paper. We now include a model where the prior precisions for task 2 are the same as those for task 1 (see “Reduced Precision Representation” in the Results section). This has been implemented using changes in precisions only, as this effectively reduces Bayesian learning to something much closer to maximum likelihood learning – and therefore tests whether or not the Bayesian aspect of the modelling is doing anything useful.

**Q3.2** I also feel the data are a little
“over-analysed”, whilst important things are missing. The discussion
lists a number of open questions and limitations, which is honest, but
does leave the reader wondering what can be inferred from the results.
With these things in mind, on balance, I felt a little underwhelmed with
the paper. There are good things there, and some of the issues listed
below can be straightforwardly addressed, but I think the paper would
gain a lot more from running an additional experiment with multivariate
subspaces.

We have designed a multivariate subspace experiment and will shortly be piloting it. Whilst we agree that results from this experiment would be wonderful to include, we feel that the current paper already contains substantial empirical and intellectual content.

Major issues

**Q3.3** Apart from the form, no mention is made of open science practices
in the actual manuscript. I strongly urge the
authors to make their data, materials, and analyses scripts
available.

Our data and software are now available on Github, as described in the “Data and Software Archive” section. See <https://github.com/wpennyUEA/subspace>

**Q3.4** The main analysis showing the benefit of shared subspaces consists
of two separate t-tests. However, showing that one test is not
significant, and another is, is not itself a good test or indication
of a significant interaction effect, which is really what the
hypothesis is about (a larger increase in performance for shared vs
non-shared subspaces). the authors should minimally show that this
interaction is directly significant.

This is a fair point and we now report the interaction. Overall, this is not significant. Breaking this down into subspace type, as proposed in Q3.5, we find the interaction is not significant for the Add subspace, but is for the Sub subspace.

**Q3.5** Is collapsing over subspaces warranted? All analyses collapse over
the subspace type (additive vs subtractive), which makes sense given
the goals of the study, but not so much given what we know about
human function learning (which shows a clear benefit of positive
functions (additive) vs negative functions (the subtractive subspace
can be seen as a combination of a positive and negative function).
The authors should at least show the performance separately for the
additive and subtractive subspaces (and the second task also as a
function of whether the subspace was additive or subtractive in the
first task). I think this may highlight important deviations between
human behaviour and the models (which won’t inherently care whether
the subspace is additive or subtractive).

We now include a section in the discussion (“Learning accuracy across mappings”) in which the literature on human function learning is briefly discussed – as this literature seems to focus entirely on one-dimensional functions we find it difficult to relate to the current paper.

We have now reorganised our behavioural analyses, and model simulations, to include separate reporting of findings for Add and Sub subspaces. As you suspected, there are differences – not in the signs of the effects but whether or not the effects were significant.

**Q3.6** There is a proliferation of analyses, which can lead to increases
false positives. I think the main important analyses can be
conducted with a single (larger) factorial ANOVA model, with task (1
or 2), subspace task 1 (additve, subtractive), subspace similarity
task 2 (same, different), and block as factors. The main effect of
interest would be the “task” by “subspace similarity task 2”
interaction, but the analysis will control for a number of other
factors, which might be of interest as well.

We have now greatly reduced the number of analyses we present. We have removed the subspace-by-block interactions, effect of learner (i.e. good versus bad), and same-subspace learner-by-block interaction.

We now present only (i) correlations in accuracy between task 1 and task 2 (a) overall, and separately for the (b) Add and (c) Sub subspace, (ii) two way ANOVAs with dependent variable accuracy and independent factors of task and (same/different) subspace (a) overall, and separately for the (b) Add and (c) Sub subspace.

Overall, the empirical subspace effects are a significant difference in correlation for the Add subspace, and a significant interaction for the Sub subspace.

**Q3.7** I didn’t find the analyses involving a median split on good vs bad
performers that informative. Given the bounded scale of the
dependent variable, it is not so strange that those who initially do
well don’t improve as much as those who initially perform poorly.

We have now removed this analysis.

**Q3.8** I imagine the use of online batch learning for the two parameter
sharing models was used in order to allow for the model to flexibly
choose a “right” level of transfer for the soft sharing version.
That, as well as the “check solution is OK or increase the variance
of the prior” seems like a bit of a “hacky” solution to me. At least
as a model of human behaviour, it would predict no performance
increase within blocks, which seems unlikely to me. So that begs the
question whether (apart from being able to transfer or not depending
on the task similarity), the soft parameter sharing model is really
a plausible model of human learning. The way soft parameter sharing
works seems rather reminiscent of things like Dirichlet processes
and Anderson’s model of rational category learning, where a new
representation for a task would be learned whenever it doesn’t fit
an already learned representation very well. If a mixture approach
is taken, where the new task is either from the posterior
distribution of the previous task, or drawn from a more general
prior suitable for new tasks, I think a more principled approach
could be taken, where the posterior over mixture components defines
the weight between transfer and no-transfer. This would have the
benefit of being both “properly Bayesian” and allowing for a fully
online model.

We agree that the “check solution is OK or increase the variance
of the prior” seems a bit hacky and have decided to abandon this approach.

In its place, we now focus more on the basic framework of Sequential Bayesian Learning which has been shown to work well on scaled up problems under the name Elastic Weight Consolidation [11]. Within this, we consider three variants (i) a minimal capacity model, (ii) an increased capacity model and (iii) a reduced precision representation, with only (i) doing a reasonable job of replicating the empirical subspace findings. See new results sections for details.

Having abandoned our hacky SPS approach, we also took the opportunity to re-engineer the output subnetworks which were previously instantiated using an RBF approach. We were unhappy with this because, although learning was fast, the basis function centres have to be rather hand-crafted and RBFs don’t scale well. We therefore replaced them with GELU units (see section on “Value Network”) , thus making the overall model more similar to multitask/transfer models using deep neural nets [11].

Finally, the possibility of using mixture priors over neural net parameters is referred to in a new Discussion section (“Discrepancies between model and behavioural data”).

**Q3.9** The comparison between the two parameter sharing models and the RBF
seems rather unfair. The first two are trained in an offline batch
manner, optimizing the learning rates for each batch, while the last
is trained in an online manner, with a fixed (arbitrary?) learning
rate. Given the many differences, any comparison in model
performance is difficult. If the interest is in transfer vs no
transfer, why not stick to the same general model as the parameter
sharing models (using a completely “refreshed” prior for the second
task). If the interest is in a difference in representation, why not
allow the RBF model to use soft and hard parameter sharing?
Personally, I’d like to see the results of all six models, and all
trained in the same way (preferably online). At the moment, no clear
conclusions can be drawn from comparing RBF to the other two models.

We have now removed the RBF models. We now include a model in which the prior is refreshed (in the sense of having the same precision as at the beginning of Task 1) – see answer to Q3.1.

Minor issues

**Q3.10** line 128. What was the duration of break between blocks supposed to
achieve?

We had an orthogonal hypothesis that the duration of breaks (short versus long) might influence learning. This effect is not reported in the current paper as there are too many other issues to focus on.

**Q3.11** line 364: “overtime” -> “over time”

A whole section of text including this has been removed.

**Q3.12** Appendix A.3.2 and A.3.3 I don’t think the ANOVA on the models with
“good” vs “bad” learner is overly meaningful. Especially for the
RBF, which uses the same parameters throughout, good vs bad may
depend somewhat on the input, but I doubt there is a meaningful
distinction. For the other two models, which depend on some
randomness in priors, the distinction may be larger (hence the
differences found), but then may be due to rather uninteresting
differences, rather than anything meaningful.

We have now removed the “good” versus “bad” learner data analysis.

**Q3.13** Appendix B.3 “We therefore infer these effects to be collinear and,
with the present data, we cannot tell which of these factors is
driving the subspace effect.” There are agreed upon measures for
collinearity (which depend on relations between the independent
variables/predictors), such as the tolerance or variance inflation
factor. The finding that effects or not significant if both
predictors are included in a model is likely due to collinearity,
but it is not a suitable test of it.

We have now added the Variance Inflation Factor (VIF) calculation. The two predictors are moderately collinear with a VIF of 1.751.
