# Supplementary material for: Multitask learning over shared subspaces
Source: PLoS Comput Biol. 2021 Jul 6;17(7):e1009092. doi: 10.1371/journal.pcbi.1009092 (PMC8284664; doi:10.1371/journal.pcbi.1009092)
Supplement: Attachment — Submitted filename: response-to-reviewers.docx [file pcbi.1009092.s003.docx]

# Reviewer 2

I thank the authors for the thorough revision!

Regarding connections with the literature:

Thanks for the discussion of:
* Structure learning
* Elemental vs. configural learning
* Multitasking NNs
I think it will be very helpful for people interested in the issue of transfer learning to be able to connect with this literature.

**Q2.1** If I may suggest moving these bits from the Intro to the Discussion under a larger "Related Work" section, in which authors highlight after each one how their approach advances or relates to each line of work. E.g, move the short para on page 7 to the Discussion, and say that the author's approach is one way to resolve the issue of elemental learning being insufficient for nonlinear functions, and the configural learning being, indeed, very costly. That way the focus will stay on the authors' main contribution. It felt like this comment (creating an explicit Related Work section) may apply to other parts of the Discussion as well.

We have now moved these sections to the discussion (incorporating the text on page 7) as suggested.

I also appreciated the discussion of how the issue of linear/non-linear mappings interact with the results, and agree that it is hard to disentangle from an order effect with the current design.

Regarding results:

The behavioral results are much easier to parse, thank you!

**Q2.2** In the Discussion when summarizing results, authors may want to note that the performance boost by a shared subspace is task-dependent, in that it holds for Addition, but not Subtraction. You can leave it to future work as to why that may be the case. But it seems important to be precise here, especially because it's a result that points to possible changes to the model.

We have now rewritten the beginning of the discussion to summarize the patter of transfer effects more precisely – with separate statements for the Add and Sub subspaces.

**Q2.3** I'm also now realizing after seeing the new plots that the correlation in performance between Task 1 and Task 2 happens only for Add (Figure 4, page 15, paras 1 and 2 -- this result is not shown in the figure, but mentioned in the text). This is perhaps inconsistent with the result in Figure 6, in which the behavioral boost attributed to the shared subspace happens for Sub, but not Add. What might be the explanation there?

These observations are correct. We have now updated Figures 4 and 5 to separately plot the results for the Sub and Add subspaces. This should make it clearer that the correlation effect is only observed for the Add subspace and the mean effect (boost) is only observed for the Sub subspace. We don’t know why the boost is not observed for Add. We’ve added the following text to the correlation section of the results:

“We do not know why there would be no significant correlations for the Sub subspace but note that the variance of Task 1 accuracies is significantly lower for Sub1 than Add1 (Std Dev=0.06 for Sub, 0.11 for Add, Levene’s test F(1,78)=10.94, p=0.001). Generally, lower variances make it more difficult to detect co-variances/correlations.”.

**Q2.4** In the learning curves, perhaps use a vertical line to mark the start of Task 2 between Blocks 5 and 6?

We have included a vertical line in the new version (Figure 6).

**Q2.5** Finally, for the section titled "Discrepancies between model and behavioral data": authors may want to rename it to something more positive like, "Future Modelling Work".

Thanks ! – we’ve renamed this section.

# Reviewer 3

**Q3.1**

Review of “Multitask Learning over Shared Subspaces”

Summary

This study presents an experiment on transfer between tasks with a
shared or non-shared feature subspace, as well as three artifical neural
network models with Sequential Bayesian Learning. The experiment shows a
benefit in performance when tasks share a subspace, and the modeling
results show that a “minimal capacity” ANN with SBL matches human
performance better than an “increased capacity” ANN and a model with
“reduced precision” (which increases the variance of the prior in the
transfer task).

Evaluation

In response to the previous review round, the authors did an extensive
revision of the manuscripts and particularly the modelling. The
statistical analysis of the behavioural results is much cleaner now. The
new modelling is not really comparable to that of the previous
submission, but I like that all models presented derive from a single
framework. However, I find the difference between the restricted and
increased capacity model less intuitive as a manipulation of learning
transfer. Perhaps more importantly, the restricted capacity model with
SBL matches the transfer patterns not by offering benefit for a shared
subspace task, but rather a detriment for a non-shared subspace task. As
an account of learning transfer, that seems rather disappointing.

Many thanks for this very useful feedback.

A3.1 Re: “the restricted capacity model with SBL matches the transfer patterns not by offering benefit for a shared subspace task, but rather a detriment for a non-shared subspace task”

This is an important observation that we have addressed in the new submission. We now present new modelling results after having made a single change to the simulations. This was to use linear units (rather than GeLU units) in the Task 2 output subnetworks – motivated by the fact that the mapping from subspace to output is linear for Task 2 mappings. This change results in a clear and significant benefit for the shared subspace tasks (as well as maintaining a detriment for non-shared subspace tasks).

See our response to A3.4 for more details.

**Q3.2**. The analysis of the behavioural results is much tighter now. But the
results of the two mixed ANOVAs are difficult to interpret without
seeing the data separately for the different types (additive vs
subtractive). The plots (Fig 4 and 5) should really reflect the new
analyses, and not show the data aggregated over the types.

A3.2 We have now plotted results separately for the Add and Sub subspaces in the new Figures 4 and 5.

**Q3.3**. The difference between the restricted and increased capacity model
can be more clearly described. What is the effect of setting the
number of hidden units in the first hidden layer to 1 vs 2 on the
solutions that can be achieved? As the results show, it seems
crucial to replicating worse performance in the different subspace
conditions that there is one hidden unit in the first layer, but as
someone who doesn’t work with ANNs often, I find it difficult to
interpret the meaning of this, especially since the reduced and
increased ANNs both use SBL. Is there perhaps a substantial
difference in the precision of the posterior distribution over the
connection weights, such that the increased capacity model can more
easily overcome a “wrong” prior? Or is something else going on?

A3.3 We have now added reports of the posterior precisions for the minimal capacity, increased capacity and reduced precision models. For the increased capacity model we now write:

“Quantitatively, the standard deviations of hidden unit parameters were 19 times smaller in the posterior (after learning Task 1) than the prior (before Task 1). The figure of 19 is an average over all weights in both hidden units and over both Add and Sub Tasks. The precisions were thus $19^2=361$ times higher in the posterior (after Task 1) than the prior (before Task 1). This is a smaller increase than for the minimal capacity network, thus rendering Task 2 solutions somewhat less constrained to be similar to Task 1 solutions (see last row of equation 18). However, we expect that the main factor in the loss of transfer effects is the increased representational capacity of the network (the required subspace for the Task 2 mapping can be implemented by either hidden unit or distributed over both).”

The equivalent section for the minimal capacity model is:

“Quantitatively, the standard deviations of hidden unit parameters were 24 times smaller in the posterior (after learning Task 1) than the prior (before Task 1). The figure of 24 is an average over all weights in the hidden unit and over both Add and Sub Tasks. The precisions were thus $24^2=576$ times higher in the posterior (after Task 1) than the prior (before Task 1). As the prior (before Task 2) is set to the posterior (after Task 1) this strongly constrains the Task 2 solution to be close to the Task 1 solution (see last term in last row of equation 18).”

The equivalent section for the reduced precision model is:

“(quantitatively, the precision variable, $\lambda_{j-1}$, in the last row of equation 18) is 576 times smaller than for the minimal capacity network - see above section). This results in Task 2 solutions being only very weakly constrained to be similar to Task 1 solutions, thereby eliminating the subspace effect.”

**Q3.4**. I’m not convinced the new models match the behavioural patterns that
well. The “minimal capacity” model shows not so much a benefit of
same subspace, but a detriment of different subspace. The behaviour
of participants, on the other hand, seems to indicate a benefit of
shared subspace (especially in the Sub condition, where performance
is immediately better in Task 2 than Task 1). Model performance in
Task 2 never exceeds that of Task 1. While the differences between
the model and human behaviour are acknowledged in the Discussion, I
found the explanations not that convincing. In particular, if
participants find the linearly separable tasks easier, that seems to
go against a transfer of a learned “shared subspace” representation.

A3.4 Re: “The “minimal capacity” model shows not so much a benefit of
same subspace, but a detriment of different subspace”.

We have now re-run our simulations using linear hidden units in the Task2 output sub-networks (see A3.1 above) and find that the minimal capacity model does now show a benefit of same subspace (in addition to a detriment of different subspace). This is reported in the Minimal Capacity Network section under Sequential Bayesian Learning over Tasks and also in the section on Sequential Bayesian Learning over Blocks and Tasks. The effect can also be seen in Figure 6 (right panel). Other results subsections have also been updated in light of this change.

Re: “if participants find the linearly separable tasks easier, that seems to
go against a transfer of a learned “shared subspace” representation”.

Simulated participants only find the linearly separable tasks easier if they lie in the shared subspace (see Figure 6). Whilst there are still discrepancies between model and behavioural trajectories, potential routes forward for ameliorating them are proposed in the Discussion.

**Q3.5**. Model performance was matched to behaviour by changing the maximum
number of starts of the optimization routine. It is not immediately
obvious to me where this applies, but I guess its the number of
reductions of the step size in the fminbnd.m routine? Setting this
number at a low value makes it likely that the result is a local,
rather than global, maximum. Why is this chosen to effectively
reduce model performance, rather than other possibilities
(e.g. introducing randomness in the responses, reducing the
effective learning rate by increasing the precision of the prior,
etc). Compared to these latter possibilities, the “maxstart”
parameter seems a rather “hacky” choice. This is of course
subjective, but this particular setting of a numerical optimization
routine seems quite far removed from anything that might be
plausibly differ between participants. Can more justification be
given for this choice? Alternatively, if this variation in this
parameter is just a robustness check, and nothing of theoretical
interest, perhaps state this clearly.

Estimation of neural network parameters used a within-block multistart optimisation procedure as described in the “MAP Estimation” section. Essentially, if a satisfactory solution is not found (i.e. the local maximum is not good enough) the optimisation is run again from a different initial parameter setting – a different sample from the prior.

To clarify this, we have added “This parameter helps the optimiser avoid local maxima by restarting the optimisation with a different initialisation” to the Results section, and a reference to the multi-start optimisation literature in the MAP Estimation section.

The “maxstart” parameter is nothing to do with the fminbnd routine, but simply the number of optimisation runs allowed per block.

We also added the following text to the Discussion:

“The model we have presented employs a within-block multistart optimisation procedure such that if estimated parameters do not provide a sufficiently good solution, the estimation is repeated, with a total of maxstarts=3 model fits allowed per block. This serial model fitting process is biologically implausible but could potentially be implemented using parallel architecture and may fit in with evidence that up to three or four decision making strategies can be simultaneously updated and monitored [29]. One possibility for future modelling, however, would be to use a moving window of samples to which the model is fitted, rather than splitting the samples into non-overlapping blocks. This would remove the "blockiness" of the results referred to above, and the inherent stochasticity of the approach may remove the need for multistart optimisation. Such an approach has been used to good effect in recent work on dynamical models [30]”

We also emphasise that no model parameters were tuned to individual participants:

“We emphasise that no model parameters were specifically tuned to the particular subspace (Add or Sub) or to individual subject data. The models were simply provided with the above parameter settings, and the same stimuli and reward functions provided to the participants”.

Minor issues

**Q3.6** section “Sequential Bayesian Learning over Blocks and Tasks”. Which
model is used here? I’m assuming the restricted capacity model, but
this should be stated clearly.

We now state this more clearly: “We now report results using Sequential Bayesian Learning over blocks and tasks for the minimal capacity model”.

**Q3.7** Can Figure 6 and 7 be combined in a single figure, so its easier to
judge the closeness of behaviour and model predictions?

We now include these in a single Figure (Figure 6).

**Q3.8** line 576. Subjects may not have been told that they would be tested
again on Task 1, but they were also not told the contrary. Why would
they assume they would not encounter the first task again?

This is a reasonable point. We have now updated the relevant text as follows:

“In our behavioural experiments participants only performed Task 1 once, and were not told that they would be performing it again. This may have led to an expectation that they would not need to retain Task 1 representations. If this were the case, an increased capacity network would not be needed; a minimal capacity representation would suffice that could be overwritten during the second task. However, we do not know the subject’s expectations because they were not explicitly manipulated. This does however motivate a future experiment. If participants were to be told that they will be tested again on Task 1, after learning Task 2, then the prediction is that we will not see negative transfer effects when Task 2 is in a different subspace to Task 1. Such an experiment would speak to a distinction in the neural network literature \cite{parisi19} in which two of the major approaches in the area of transfer/continual learning are to use (i) dynamic architectures in which new representational capacity is added for each new task to be performed and (ii) regularisation approaches in which a fixed architecture is used but regularisation prevents forgetting.”

**Q3.9** line 628: “non-linearly separable categories are easier to learn.”
Easier than what? And do the authors mean “linearly separable
categories”?

We’ve now added “than linearly separable ones”.

**Q3.10** Appendix C.2: How was it determined that an open-ended self-report
of a participants’ strategy matched their actual strategy? This
would be quite a complicated thing to determine, so more information
on the procedure is needed.

The text in the appendix now appears as follows (new text in red):

“We first define a declarative learner as a subject who correctly declared their strategy for the first task. This was based on the participant's response to the open-ended question posed after each task: "How did you approach the task?". A correct declaration of strategy was inferred if their verbal report contained the following phrase (i) for Sub1 "if the number of pie slices is the same/similar in both pies, then choose Sun" or , (ii) Add1 "if the total number of slices on the left and right makes a complete pie, then choose Sun". Phrases that were deemed logically or semantically equivalent were also assessed positively. All assessments were made by author NM.”
